# On the bioherbicide potential of *Ulex europaeus* and *Cytisus scoparius*: Profiles of volatile organic compounds and their phytotoxic effects

**DOI:** 10.1371/journal.pone.0205997

**Published:** 2018-10-29

**Authors:** María Pardo-Muras, Carolina G. Puig, Antonio López-Nogueira, Carlos Cavaleiro, Nuria Pedrol

**Affiliations:** 1 Department of Plant Biology and Soil Science, University of Vigo, Vigo, Spain; 2 Faculty of Pharmacy and CNC, University of Coimbra, Coimbra, Portugal; Instituto Agricultura Sostenible, SPAIN

## Abstract

The phytotoxic potential of the legume shrubs *Ulex europaeus* L. (gorse) and *Cytisus scoparius* (L.) Link. (Scotch broom) is studied in this work for the first time. On the basis of their richness in active principles, the previous evidence of biological activity, and the abundance of biomass in their native range and invaded areas, a question arose: can *U*. *europaeus* and *C*. *scoparius* be considered as potential sources of natural herbicides for sustainable agriculture? By means of volatile bioassays, the flowering fresh plant material of both shrub species was shown to produce and emit volatile organic compounds (VOCs) able to inhibit the germination and/or early growth of two agricultural weeds: *Amaranthus retroflexus* and *Digitaria sanguinalis*. Novel complete VOCs profiles from the volatile extracts of the shrub species were obtained by GC and GC/MS. A total of 20 compounds were identified from *U*. *europaeus* flowering biomass, theaspirane and eugenol, among others, being described in gorse for the first instance. The chemical profile of *C*. *scoparius* yielded 28 compounds and was rich in oxygenated monoterpenes such as terpinen-4-ol, verbenol, *α-*terpineol, and verbenone, which were also identified in this species for the first time. Using dose-response bioassays with pure compounds, these VOCs were argued to be involved in the phytotoxicity observed for the plant materials, even at very low concentrations. The phytotoxic effects were predominantly irreversible, particularly for *D*. *sanguinalis*, since the seeds exposed to the VOCs produced damaged seedlings, were unable to recover germination capacity after removing the phytotoxin or, when recovered, produced unviable seedlings. Our results extend the interest of the abundant *U*. *europaeus* and *C*. *scoparius* for the obtention of natural products with bioherbicide potential, or to be used as allelopathic biomass in the development of new sustainable agricultural practices.

## Introduction

Legume species have major relevance in agriculture and agroforestry worldwide. They are vital components in ecosystems for their role as atmospheric nitrogen fixers through their association with *Rhizobium* bacteria. From the Fabaceae family, the perennial shrubs *Ulex europaeus* L. (gorse) and *Cytisus scoparius* (L.) Link. (Scotch broom) are native to the Atlantic region. Gorse is native to the western coast of continental Europe and the British Isles, whereas Scotch broom is widely distributed all across Europe. The Atlantic shrubland is dominant in the native range [1–3]. Outside their natural distribution range, *U*. *europaeus* and *C*. *scoparius* are considered highly invasive weeds [4–6]. In fact, gorse is considered one of the 100 worst invasive species in the world [7].

The invasiveness of these species is closely linked to their ability to sprout and regenerate rapidly, the difficulty for their eradication, a high seed production, and their fire-resistance [8, 9]. However, other factors underlying the competitive ability of plants are also key mechanisms to establish successfully, including interference against other species. One of these mechanisms modeled by evolution is *allelopathy* [10, 11], which has gained significance in recent years for the explanation of plant invasion success. The phenomenon of allelopathy refers to ‘any direct or indirect effect of one plant on other plants through the release of bioactive compounds (named allelochemicals) by volatilization, leaching, exudation from roots or decomposition of plant residues’ [10].

Both gorse and Scotch broom have been appraised from ancient times for their high content in bioactive compounds. Several species of the genus *Cytisus* have been used in folk medicine ([12, 13] and references therein). In particular, *C*. *scoparius* extracts based on organic solvents have shown antifungal [12], antimicrobial [14] and antioxidant activities, e.g., [12, 15]. Also, *U*. *europaeus* has been studied for its antioxidant [16] and antifungal [17, 18] properties. However, can we consider these species to be allelopathic? Some recent evidence supports it. Grove et al. [19] argued allelopathy as the possible mechanism of *C*. *scoparius* for competing intensively with native vegetation, thus reducing recruitment of seedlings and growth of understory species in open forest areas. Also, López-Nogueira et al. [20] suggested that the legume species from the Atlantic shrubland are highly competitive also in the native range, being able to stop the spread of invasive tree species through allelopathy. The emission of volatile organic compounds (VOCs) from *U*. *europaeus* in the different phenological stages throughout the year has been studied by some authors [16, 21, 22] thus demonstrating the continuous release of potentially bioactive compounds from this species. VOCs are in fact allelochemicals produced and emitted by plants that play important roles in biotic interactions such as pollinator attraction or plant defense [23–25]. However, the volatile profiles of gorse and Scotch broom are as yet poorly known.

Allelopathy also plays essential roles in agroecosystems influencing weed growth and crop yield [26, 27]. For this reason, allelopathy and the allelochemicals involved are receiving notable attention as possible sustainable alternatives to the use of synthetic herbicides [28, 29]. Especially, VOCs have excited the greatest interest as natural herbicides [30–32]. They have been described as potent inhibitors of seed germination and growth of several plant species, e.g., [33–35]. However, they continue to be a largely untapped source of active compounds for potential use in agricultural fields. Interestingly, very recent approaches have proposed the use of allelopathic biomass from forest residues or invasive tree species as bioherbicide green manures [36, 37]. Allelopathic biomass incorporated into the soil can release genuine ‘cocktails’ of allelochemicals that could control the germination and growth of different weed species.

Then, considering previous evidence of biological activity, the richness in active principles, and the abundance of shrub biomass in our agroecosystems, a question arises: can *U*. *europaeus*, and *C*. *scoparius* be considered as potential sources of natural herbicides for sustainable agriculture? As far as we know, the potential phytotoxic effects of gorse and Scotch broom on agricultural weeds have not yet been studied.

To answer this question, the following objectives were proposed: (i) to assess the *in vitro* phytotoxic potential of volatiles emitted by flowering biomass of each shrub species, on the germination and early growth of two agricultural weed species; (ii) by GC and GC/MS, to determine the chemical profile of the volatile extracts of flowers and flowering branches of both shrub species; (iii) to identify which VOCs are potentially involved in the phytotoxicity observed, by means of dose-response bioassays of isolated compounds on the germination and early growth of the two agricultural weeds; and (iv) to test the reversibility of the effects of the most phytotoxic VOCs.

## Materials and methods

### Plant material

Flowering branches of *U*. *europaeus* and *C*. *scoparius* were collected over April and May 2014 at different locations, the first species in Cabo Home (Galicia, NW Spain, 42°16'08.9" N 8°51'38.0" W), and the latter in the vicinity of the University of Vigo (Galicia, NW Spain, 42°09'56.0" N 8°41'04.7" W). No specific permission was required for these locations and plant species, since both gorse and Scotch broom are regularly thinned to clean paths and viewpoints, and they quickly regenerate. Fresh plant material was taken immediately to the laboratory for further processing.

Once in the laboratory, the pool of plant material for each species was separated into two portions: one for *in vitro* volatile bioassays, and the other one for the extraction and chemical analysis of VOCs. All the volatile bioassays and analyses were carried out for flowering branches and also for flowers alone.

### Naturally emitted volatile bioassays

To assess the *in vitro* phytotoxicity of VOCs emitted by *U*. *europaeus*, and *C*. *scoparius*, a battery of bioassays was performed after Barney et al. [38]. Plant material was wrapped in a sterile cotton gauze swab (1 mm mesh size) and introduced into a 1-L hermetic glass chamber, hanging from the top for preventing physical contact between seeds of the target species and the donor plant material, but allowing VOCs to flow inside the chamber atmosphere (S1 Fig). Treatments consisted of fresh plant material equivalent to 2 g of dry weight of green flowering branches (flowers, leaves and shoots, and thorns in the case of *U*. *europaeus*) or flowers alone [i.e., 6.0 and 7.85 or 6.45 and 12.7 g fw, of flowering green branches and flowers of *U*. *europaeus* or *C scoparius*, respectively]. Control treatment consisted of cotton gauze swab containing pieces of drinking straws at the same volume as fresh plant material.

*Amaranthus retroflexus* L. (redroot pigweed) and *Digitaria sanguinalis* (L.) Scop. (large crabgrass) from Herbiseed (Twyford, England, UK) were used as representative dicotyledon and monocotyledon highly competitive weed species [36]. *Amaranthus retroflexus* seeds were previously synchronized by soaking in distilled water for 15 days at 4°C and then air dried, whereas *D*. *sanguinalis* seeds were placed under light for 56 days at 4°C.

For germination bioassays, twenty-five seeds per chamber were placed on a filter paper layer wetted with 4 ml of distilled water; then, the chamber was hermetically closed and incubated at 27°C in the dark. This way, the seeds were continuously exposed to the VOCs emitted by the plant material over the time assayed. The number of germinated seeds (rupture of seed coats and the emergence of radicle ≥1 mm [39]) was counted every 12 h for *A*. *retroflexus*, and every 24 h for *D*. *sanguinalis*, until no further germination events were observed in the control. The total percentage of germinated seeds (Gt) and the coefficient of the rate of germination (CRG) were calculated after Chiapusio et al. [40] and De Bertoldi et al. [41]. For early growth bioassays, fifteen pre-germinated seeds (root length between 1 and 3 mm [39]) were used under the same conditions as for germination bioassays. Root and shoot lengths of the pre-germinated seeds were measured after 48 h, and a morpho-anatomical description of the seedlings was made by using a Nikon SMZ 1500 electronic magnifier (Nikon, Melville, NY, USA). Then, root and shoot biomass per chamber were obtained by drying the fresh material at 70°C for 72 h. For each treatment and target species, four replicates were carried out.

#### Extraction and chemical analyses of VOCs

Volatile extracts from flowering branches or flowers of *U*. *europaeus* or *C*. *scoparius* were obtained by continuous water distillation/solvent extraction for four h, using a Likens-Nickerson type apparatus [42] and *n*-pentane as solvent [43].

#### Gas chromatography and gas chromatography/mass spectrometry analyses

Analytical GC was carried out in a Hewlett-Packard 6890 (Agilent Technologies, Palo Alto, CA, USA) gas chromatograph with an HP GC ChemStation Rev. A.05.04 data handling system equipped with a single injector and two flame ionization detection (FID) system. A GraphPad divider (Agilent Technologies, part no. 5021–7148) was used for simultaneous sampling to two Supelco (Supelco, Bellefonte, PA, USA) fused silica capillary columns with different stationary phases: SPB-1 (polydimethylsiloxane 30 m × 0.20 mm i.d., film thickness 0.20 μm), and SupelcoWax-10 (polyethylene glycol 30 m × 0.20 mm i.d., film thickness 0.20 μm). Oven temperature program: 70–220°C (3°C min^-1^), 220°C (15 min); injector temperature: 250°C; carrier gas: helium, adjusted to a linear velocity of 30 cm s^-1^; splitting ratio 1:40; detectors temperature: 250°C. GC/MS was carried out in a Hewlett-Packard 6890 gas chromatograph fitted with an HP1 fused silica column (polydimethylsiloxane 30 m × 0.25 mm i.d., film thickness 0.25 μm), interfaced with a Hewlett-Packard mass selective detector 5973 (Agilent Technologies) operated by HP Enhanced ChemStation software version A.03.00.GC, with parameters as described above; interface temperature: 250°C; MS source temperature: 230°C; MS quadrupole temperature: 150°C; ionization energy: 70 eV; ionization current: 60 μA; scan range: 35–350 units; scans s^-1^: 4.51.

#### Identification of VOCs from flowering branches and flowers of gorse and Scotch broom

Compounds were identified by their GC retention indices on both SPB-1 and SupelcoWax-10 columns and from their mass spectra. Retention indices, calculated by linear interpolation relative to retention times of C8–C23 of *n*-alkanes, were compared with those of reference samples included in our laboratory database (C.E.F. / Faculty of Pharmacy, University of Coimbra). Acquired mass spectra were compared with reference spectra from the laboratory database, Wiley / NIST library [44] and literature data [45, 46]. Relative amounts of individual components were calculated based on GC raw data areas without FID response factor correction.

### Phytotoxicity dose-response bioassays of some identified isolated VOCs

Twelve volatile compounds present in the chemical profiles of the flowering branches and the flowers of *U*. *europaeus* and *C*. *scoparius* were selected by (i) previous evidence of phytotoxicity in the literature, (ii) the commercial availability, and (iii) their abundance in the analyzed plant material. Four oxygenated monoterpenes (linalool, terpinen-4-ol, *α-*terpineol and verbenone), one benzenoid compound (eugenol), one oxygenated norisoprenoid (theaspirane) and six aliphatic compounds (*n-*nonadecene, *n-*eicosane, *n-*heneicosane, *n-*docosane, *n-*tricosane and *n-*tetracosane) were tested on the germination and early growth of *A*. *retroflexus*, and *D*. *sanguinalis*.

Chemicals were purchased from Sigma–Aldrich Chemical (St. Louis, MO, USA) and used without further purification. For dose-response bioassays, a filter paper strip was fixed to the top lid of the plate, and the corresponding quantity of compound was added with a micropipette; in this way, the compound was only in aerial contact with the target seeds [34]. Each compound was tested at 0, 0.5, 1, 1.5 and 2 μl corresponding to concentrations of 0, 6.25, 12.5, 18.75 and 25 ppm in the sealed Petri dish atmosphere, respectively. The solid aliphatic compounds were dissolved in pentane for their use; dose calculations were done basing on their density values, ranging between 0.778 and 0.797g·ml^-1^, and an average density of 0.79 g·ml^-1^ was assumed. For these aliphatic compounds, the corresponding volume of pentane was used for the control treatment. Pentane was let to evaporate before closing the Petri dish.

Germination and early growth bioassays were performed in Petri dishes (9 cm diameter) sealed with Parafilm under the same conditions described for the *in vitro* volatile bioassays. For each compound, concentration and target species, four replicates were carried out.

### Reversibility bioassays of the phytotoxic isolated VOCs

The viability of the non-germinated seeds resulting from the phytotoxicity dose-response bioassays was tested. Those compounds that inhibited the germination of at least ten target seeds per replicate were selected. Non-germinated seeds were incubated in 6-well dishes, at a rate of 10 seeds per well placed on a filter paper layer wetted with 750 μl of distilled water. Ten non-pretreated seeds for each species were used as control treatment. Seeds were incubated under the same conditions described for the dose-response bioassays. The total percentage of germinated seeds (Gt) was calculated after 20 days, and the morpho-anatomical description of the obtained seedlings was made as described above.

### Statistical analyses

Replicated experiments were conducted in a completely randomized design. Data were expressed as percentages relative to the control. After testing for normality by Kolmogorov-Smirnov test and for homogeneity of variances by Levene’s test, data were analyzed by one-way ANOVA (*P* ≤ 0.05) and LSD test (*P* = 0.05) for post hoc multiple comparisons of means. In the case of heteroscedasticity, the variance was analyzed by Kruskal-Wallis *H* test and Tamhane´s *T2* for post hoc multiple comparisons. For the dose-response bioassays of identified isolated VOCs, two-way ANOVA was previously used to test the effects of the independent variables (compound and concentration) and their interactions (compound x concentration) on each measured parameter. Dose-response curves were modeled by regression analysis with mathematical models, and the most appropriate model was selected for each case after the adjusted coefficient of determination (*r*^*2*^_*adj*_). IC_50_ and IC_80_ values (concentrations required to obtain 50 and 80% inhibition, respectively) were calculated from the generated dose-response curves, because of their usefulness to compare phytotoxicity among compounds [47]. Statistical analyses were performed using the SPSS v.19 (IBM SPSS Inc., Chicago, IL, USA) software for Windows.

## Results

### Naturally emitted volatile bioassays

The flowering branches and the flowers of both shrub species produced conspicuous phytotoxic effects on the target weed species.

**Fig 1:**
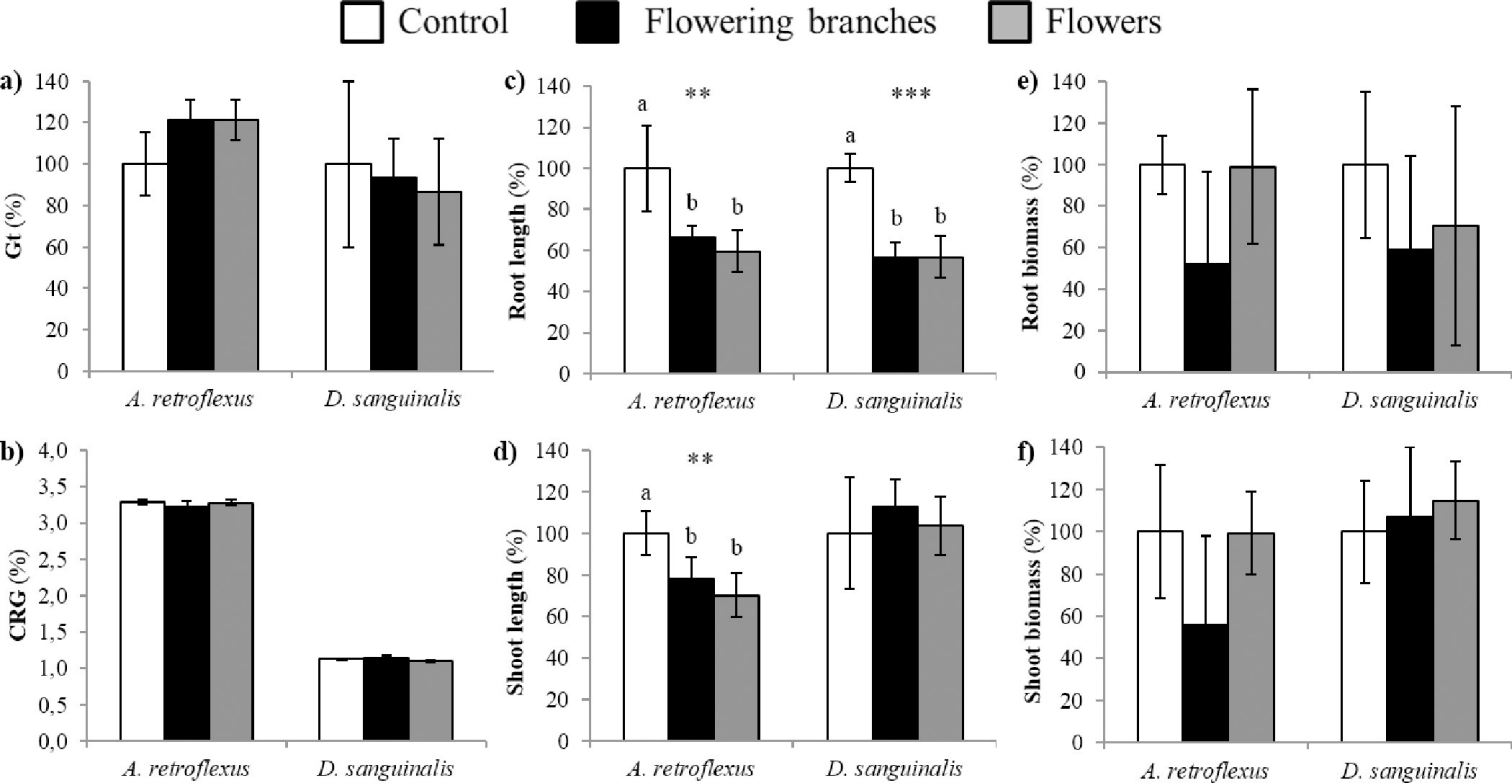
Effects of *Ulex europaeus* volatiles on the germination, growth, and biomass of two agricultural weed species (*Amaranthus retroflexus* and *Digitaria sanguinalis*). a) Total germination (Gt) b) CRG index c) root length d) shoot length e) root biomass and f) shoot biomass after the exposure to VOCs released from flowering branches and flowers of *U*. *europaeus*. Mean values are represented as percentages relative to the control. Error bars represent standard deviation (SD). For each target weed species, asterisks denote statistically significant effects of treatments at *P ≤ 0.05, **P ≤ 0.01 and ***P ≤ 0.001 (ANOVA or Kruskal-Wallis *H* test). Mean values labeled with distinct letters are significantly different at *P* ≤ 0.05 (post-hoc LSD or Tamhane`s *T2* test).

The phytotoxic effects of volatiles naturally emitted from *U*. *europaeus* fresh material are represented in Fig 1. From the analysis of variance, root and shoot lengths of *A*. *retroflexus* were very significantly affected (*P* ≤ 0.01) by the VOCs emitted from the flowering branches and the flowers of *U*. *europaeus*, thus achieving inhibitions up to ca. 40% (Fig 1C) and ca. 30% (Fig 1D) of control, respectively. In the case of *D*. *sanguinalis*, only root length was affected, with reductions about 45% of control (Fig 1C). No significant effects were observed on the total germination, CRG, or root and shoot biomass.

**Fig 2:**
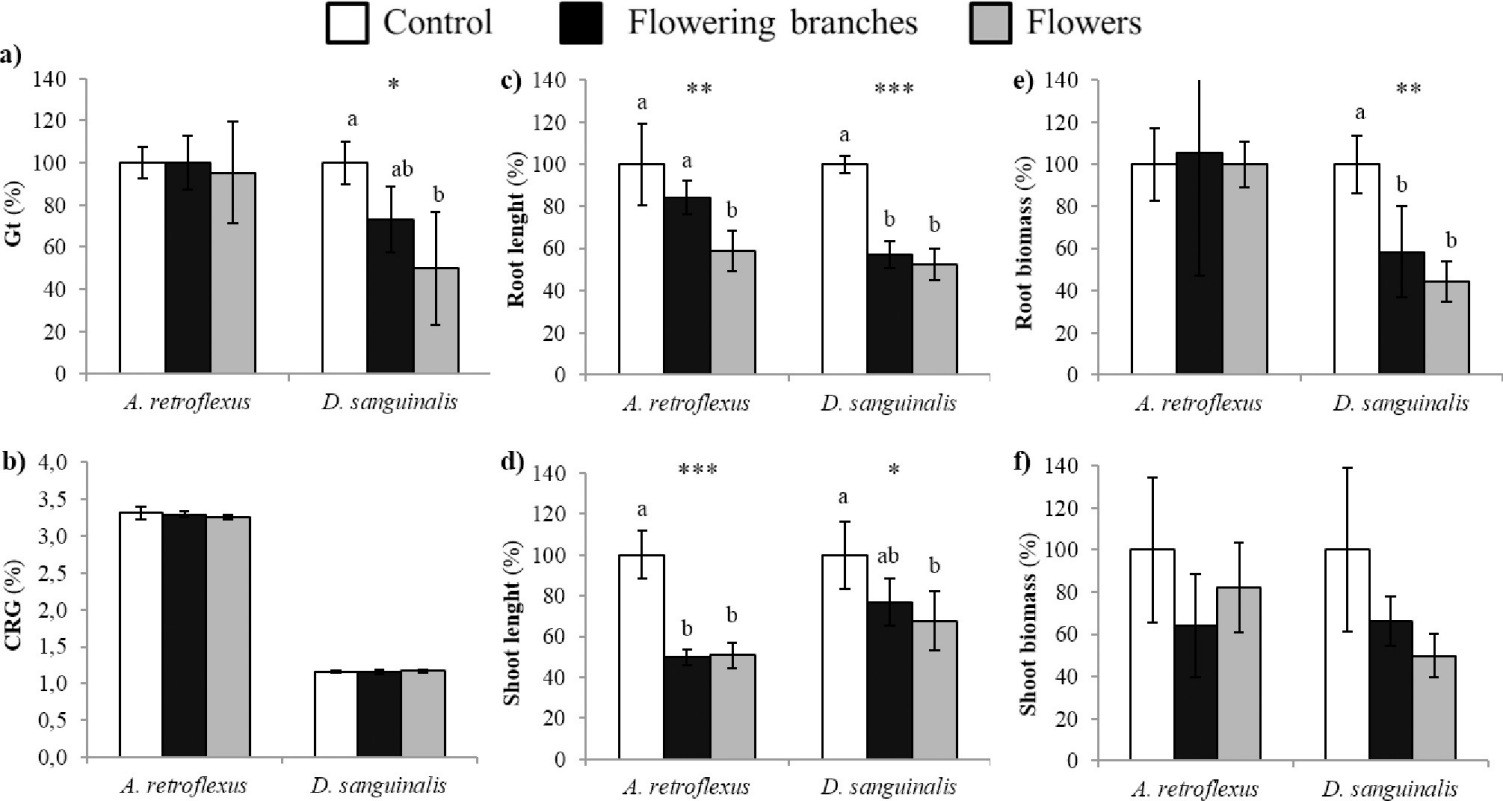
Effects of *Cytisus scoparius* volatiles on the germination, growth, and biomass of two agricultural weed species (*Amaranthus retroflexus* and *Digitaria sanguinalis*). a) Total germination (Gt) b) CRG index c) root length d) shoot length e) root biomass and f) shoot biomass after the exposure to VOCs released from flowering branches and flowers of *U*. *europaeus*. Mean values are represented as percentages relative to the control. Error bars represent standard deviation (SD). For each target weed species, asterisks denote statistically significant effects of treatments at *P ≤ 0.05, **P ≤ 0.01 and ***P ≤ 0.001 (ANOVA or Kruskal-Wallis *H* test). Mean values labeled with distinct letters are significantly different at *P* ≤ 0.05 (post-hoc LSD or Tamhane`s *T2* test).

Volatiles emitted from the fresh flowers of *C*. *scoparius* (Fig 2) significantly reduced *A*. *retroflexus* root length to 41% (*P* ≤ 0.01, Fig 2C), whereas shoot length was inhibited 50% by both flowering branches and flowers (*P* ≤ 0.001, Fig 2D); nonetheless, germination was not significantly affected. Otherwise, total germination of *D*. *sanguinalis* was reduced by 50% with respect to control (*P* ≤ 0.05, Fig 2A). *D*. *sanguinalis* also suffered inhibitions of root growth (*P* ≤ 0.001, Fig 2C) and biomass (*P* ≤ 0.01, Fig 2E) higher than 40% by both treatments, whereas shoot length was only significantly reduced to 32% by the flowers (*P* ≤ 0.05, Fig 2D). No significant effects were observed on the CRG.

### Identification of VOCs from flowering branches and flowers of gorse and Scotch broom

The extraction of volatile compounds from fresh plant material of the shrub species rendered a yellowish liquid with fresh, light liquorice odour, obtained at a mean yield of 0.06% (w/w, on a fresh mass basis) with a mean density of 0.85 g mL^-1^. Table 1 summarizes the results obtained from the GC and GC/MS analyses of the volatile extracts (from flowering branches or flowers) of *U*. *europaeus* and *C*. *scoparius*.

**Table 1:** Volatile organic compounds identified by GC and GC/MS from the volatile extracts of flowering branches and flowers of *Ulex europaeus* and *Cytisus scoparius*. Data are expressed as percentages of the total yield of the extract.

			*Ulex europaeus*		*Cytisus scoparius*	
IR^*a*^	IR^*b*^	Compound	Flowering branches	Flowers	Flowering branches	Flowers
*Monoterpene hydrocarbons*						
929	1030	*α*-pinene				0.23
1010	1187	*α*-terpinene			0.57	0.62
1046	1249	*γ*-terpinene			1.04	1.17
*Oxygenated monoterpenes*						
1148	1496	isomenthone	0.32			
1082	1543	linalool			3.08	
1158	1597	terpinen-4-ol			2.66	4.30
1122	1648	verbenol			1.58	1.50
1169	1692	*α*-terpineol			1.23	
1177	1698	verbenone			2.07	4.09
1142	1723	*p*-menth-1,5-diene-8-ol				0.58
*Sesquiterpene hydrocarbons*						
1510	1763	*β*-sesquiphellandrene				0.94
*Oxygenated sesquiterpenes*						
1545	2039	*E*-nerolidol	0.45	0.70		
1828	2126	6,10,14-trimethylpentadecanone	0.52	0.74		0.86
*Oxygenated diterpenes*						
2096	n.d.	phytol	1.85	0.92	1.90	
*Oxygenated norisoprenoids*						
1298	1487	theaspirane A	0.24			
1315	1522	theaspirane B	0.30			
*Benzenoid compounds*						
1339	2159	eugenol	0.21			
n.d.	2190	4-vinyl-2-methoxyphenol	0.22			
*Aliphatic compounds*						
1084	1393	nonanal	0.29			
959	1442	1-octen-3-ol	3.37	0.91	8.34	
n.d.	1707	8-heptadecene				0.79
1900	1900	*n*-nonadecane			1.50	1.04
2000	2000	*n*-eicosane			1.69	1.47
1697	2022	2-pentadecanone				1.18
2100	2100	*n*-heneicosane	2.46	3.06	14.75	14.28
2200	2200	*n*-docosane	0.58	1.28	3.43	2.90
n.d.	n.d.	2-heptadecanone				0.76
2300	2300	*n-*tricosane	21.29	30.01	23.02	29.56
2400	2400	*n-*tetracosane	2.50	3.99		1.54
n.d.	n.d.	lauric acid	1.57	1.10	1.66	
2500	2500	*n-*pentacosane	12.80		6.03	8.71
2600	2600	*n-*hexacosane	16.81		8.50	8.26
n.d.	n.d.	myristic acid	3.62	8.82	1.49	0.83
2700	2700	*n-*heptacosane	11.37		9.47	7.94
n.d.	n.d.	palmitic acid	13.25	12.59	5.99	2.84
**Grouped components (%)**						
Monoterpene hydrocarbons					1.61	2.02
Oxygenated monoterpenes			0.32		10.62	10.47
Sesquiterpene hydrocarbons						0.94
Oxygenated sesquiterpenes			0.97	1.44		0.86
Oxygenated diterpenes			1.85	0.92	1.90	
Oxygenated norisoprenoids			0.54			
Benzenoid compounds			0.43			
Aliphatic compounds			89.91	61.76	85.87	82.1
**Total identified**			**94.02**	**64.12**	**100**	**96.39**

IR^*a*^ retention index on SPB-1 column

IR^*b*^ retention index on a Supelcowax-10 column.

A total of 20 compounds representing 94.02% of the flowering branches volatile extract, and 11 compounds representing 64.12% of the flowers extract were identified in *U*. *europaeus*. Both extracts were qualitatively and quantitatively dominated by aliphatic compounds, *n*-tricosane being the most abundant. No monoterpene and sesquiterpene hydrocarbons were found, but some oxygenated terpenes of different complexity (e.g., isomenthone, *E*-nerolidol, phytol), oxygenated norisoprenoids (theaspirane), and benzenoids (eugenol, 4-vinyl-2-methoxyphenol) were identified.

The volatile extracts from the flowering branches and flowers of *C*. *scoparius* revealed the presence of 20 and 23 compounds constituting 100% and 96.39% of the total identified compounds, respectively (Table 1). These volatile extracts were also abundant in aliphatic compounds, thus representing more than 80% of the total composition, *n-*tricosane being the most abundant in both extracts (23.02% for the flowering branches and 29.56% for the flowers). The volatile extracts of Scotch broom also contained oxygenated monoterpenes representing ca. 10% of the total. Linalool, terpinen-4-ol, verbenol, *α*-terpineol, and verbenone were found in the flowering branches, linalool being the most abundant (3.08%) followed by terpinen-4-ol (2.66%) and verbenone (2.07%). From the flowers volatile extract, terpinen-4-ol, verbenol, verbenone, and *p*-menth-1,5-diene-8-ol were identified, with terpinen-4-ol (4.3%) and verbenone (4.09%) as majority compounds. Other identified compounds were *β*-sesquiphellandrene, 6,10,14-trimethylpentadecanone, phytol, and the monoterpene hydrocarbons *α*-pinene, *α*-terpinene, and *γ*-terpinene.

### Phytotoxicity dose-response bioassays of identified isolated VOCs

From the twelve compounds primarily selected to be tested for their phytotoxic potential on the germination of the target weed species, the analysis of variance did not yield statistically significant effects of the aliphatic compounds at their different concentrations including 0 ppm, or any significant interaction between compound and concentration (S1 and S2 Tables). Concomitantly, no visual phytotoxic effects were observed on the roots and shoots of the germinated seeds. Then, aliphatic compounds were discarded for further analysis.

All the non-aliphatic compounds assayed produced conspicuous phytotoxic effects on the target weed species. For the two target species and for all compounds, the treated pre-germinated seeds produced seedlings with curved-yellowish roots, necrotic root tips, malformed calyptra, and/or abnormal shoot growth.

The two-way ANOVA of the effects of the oxygenated monoterpenes (linalool, terpinen-4-ol, *α*-terpineol, and verbenone), the benzenoid eugenol, and the oxygenated norisoprenoid theaspirane, on the germination, root and shoot length and biomass of *A*. *retroflexus* and *D*. *sanguinalis* is shown in Table 2. The statistical analyses yielded general highly significant differences (*P* ≤ 0.001) among compounds and their concentrations and, with the exception *A*. *retroflexus* root biomass and *D*. *sanguinalis* shoot biomass, significant inter-subject effects (compound × concentration) were also observed.

**Table 2:** *P*-values obtained for the two-way ANOVA of the effects of six VOCs: Linalool, terpinen-4-ol, *α*-terpineol, verbenone, eugenol and theaspirane, the concentration assayed, and their interactions, on the germination and early growth of two agricultural weed species.

		Compound	Concentration	Compound × Concentration
*Amaranthus retroflexus*	Germination	0.000	0.000	0.000
	CRG index	0.000	0.000	0.000
	Root length	0.000	0.000	0.000
	Shoot length	0.000	0.000	0.000
	Root biomass	0.000	0.000	0.372
	Shoot biomass	0.000	0.000	0.013
*Digitaria sanguinalis*	Germination	0.000	0.000	0.000
	CRG index	0.000	0.000	0.000
	Root length	0.000	0.000	0.000
	Shoot length	0.000	0.000	0.000
	Root biomass	0.000	0.000	0.000
	Shoot biomass	0.000	0.000	0.180

Effects of the independent variables are considered significant at *P* ≤ 0.05, very significant at *P* ≤ 0.01, highly significant at *P* ≤ 0.001, and not significant at *P* > 0.05.

The best-fit equation based on the value of the adjusted coefficient of determination (*r*^*2*^_*adj*_), and the IC_50_ and IC_80_ values (in ppm) obtained from the dose-response curves (S2 and S3 Figs) are shown in Tables 3 and 4. The obtained *r*^*2*^_*adj*_ values, generally above 0.850, indicated the adequacy of the models to describe the tendency of variables and the response to different concentrations of the tested compounds, with IC_50_ and IC_80_ values mainly in the range of the concentrations assayed.

**Table 3:** Regression analyses of the dose-response effects of six VOCs on the germination and early growth of *Amaranthus retroflexus*.

		Regression equation	r^2^_adj_	IC _50_ (ppm)	IC _80_ (ppm)
Linalool	Germination	y = -3.662x + 102.0	0.915	14.20	22.39
	Root length	y = 0.091x^2^–5.685x + 102.7	0.980	11.32	23.06
	Shoot length	y = 0.138x^2^–6.808x + 102.3	0.987	9.52	21.19
	Root biomass	y = 0.092x^2^–5.313x + 99.09	0.993	11.55	o.r.
	Shoot biomass	y = 0.123x^2^–6.176x + 99.82	0.956	10.10	o.r.
Terpinen-4-ol	Germination	y = -2.231x + 104.0	0.908	24.20	*37*.*65*
	Root length	y = -2.408x + 108.0	0.687	24.09	*36*.*54*
	Shoot length	y = -1.496x + 93.84	0.680	*29*.*30*	*49*.*36*
	Root biomass	y = 0.039x^2^–3.601x + 106.9	0.804	20.24	o.r.
	Shoot biomass	y = -1.642x + 91.55	0.683	*25*.*30*	*43*.*57*
*α-*Terpineol	Germination	y = -4.354x + 102.4	0.924	12.03	18.92
	Root length	y = -3.450x + 105.2	0.832	16.00	24.69
	Shoot length	y = 0.092x^2^–5.836x + 105.2	0.928	11.57	22.78
	Root biomass	y = -3.438x + 99.54	0.901	14.40	23.13
	Shoot biomass	y = 0.105x^2^–6.195x + 103.7	0.961	10.56	20.95
Verbenone	Germination	y = 0.358x^2^–12.08x + 89.69	0.837	3.69	7.39
	Root length	y = 0.123x^2^–6.308x + 100.2	0.999	9.85	23.30
	Shoot length	y = 0.166x^2^–7.479x + 98.88	0.996	7.93	16.84
	Root biomass	y = -3.808x + 98.06	0.972	12.62	20.50
	Shoot biomass	y = 0.158x^2^–7.321x + 97.43	0.984	7.79	16.34
Eugenol	Germination	y = -0.074x^2^–1.262x + 106.4	0.787	20.37	*26*.*69*
	Root length	y = 0.108x^2^–5.281x + 99.46	0.991	12.63	o.r.
	Shoot length	y = 0.207x^2^–7.976x + 96.09	0.956	7.08	17.37
	Root biomass	y = -0.022x^2^–1.725x + 98.26	0.972	21.87	*32*.*17*
	Shoot biomass	y = 0.185x^2^–7.515x + 96.42	0.952	7.60	o.r.
Theaspirane	Germination	y = -0.064x^2^ + 1.956x + 103.9	0.336	*48*.*08*	*54*.*58*
	Root length	y = -0.015x^2^–1.077x + 105.1	0.617	*34*.*57*	*47*.*54*
	Shoot length	y = 0.018x^2^–2.221x + 103.5	0.825	o.r.	o.r.
	Root biomass	y = 0.028x^2^–2.530x + 105.7	0.795	*32*.*82*	o.r.
	Shoot biomass	y = 0.027x^2^–3.039x + 107.5	0.703	24.07	o.r.

r^2^_adj_ = adjusted coefficient of determination

IC_50_ = concentration that inhibits or reduces germination and growth at 50% of control

IC_80_ = concentration that inhibits or reduces germination and growth at 80% of control

o.r. = out of range

IC values calculated from equation overcoming the maximum assayed concentration are expressed in italics

**Table 4:** Regression analyses of the dose-response effects of six VOCs on the germination and early growth of *Digitaria sanguinalis*.

		Regression equation	r^2^_adj_	IC _50_ (ppm)	IC _80_ (ppm)
Linalool	Germination	y = -3.294x + 106.8	0.888	17.25	*26*.*35*
	Root length	y = 0.122x^2^–6.633x + 100.3	0.999	9.11	18.20
	Shoot length	y = 0.210x^2^–8.791x + 95.02	0.952	5.97	11.94
	Root biomass	y = -3.495x + 94.49	0.965	12.73	21.31
	Shoot biomass	y = 0.194x^2^–8.331x + 93.95	0.925	6.16	12.54
Terpinen-4-ol	Germination	y = 0.086x^2^–3.748x + 98.71	0.796	o.r.	o.r.
	Root length	y = -3.088x + 104.5	0.935	17.65	*27*.*36*
	Shoot length	y = -3.424x + 95.40	0.935	13.26	22.02
	Root biomass	y = -0.090x^2^–0.788x + 105.7	0.884	20.88	*26*.*79*
	Shoot biomass	y = -3.264x + 92.69	0.928	13.08	22.27
*α-*Terpineol	Germination	y = 0.150x^2^–7.436x + 101.6	0.987	8.34	16.40
	Root length	y = 0.128x^2^–5.713x + 102.2	0.931	12.82	o.r.
	Shoot length	y = 0.098x^2^–5.948x + 100.3	0.996	10.16	20.27
	Root biomass	y = 0.110x^2^–5.613x + 103.4	0.951	12.65	o.r.
	Shoot biomass	y = 0.093x^2^–5.769x + 100.6	0.996	10.57	21.25
Verbenone	Germination	y = -2.126x + 92.09	0.715	19.80	*33*.*90*
	Root length	y = 0.157x^2^–7.079x + 100.7	0.976	8.93	o.r.
	Shoot length	y = 0.196x^2^–8.447x + 97.98	0.977	6.73	13.39
	Root biomass	y = 0.104x^2^–5.830x + 103.3	0.957	11.50	o.r.
	Shoot biomass	y = 0.185x^2^–8.192x + 96.93	0.968	6.76	13.52
Eugenol	Germination	y = -0.101x^2^–0.705x + 99.09	0.993	18.83	24.71
	Root length	y = 0.239x^2^–8.824x + 93.25	0.887	5.82	12.60
	Shoot length	y = 0.295x^2^–10.62x + 91.89	0.877	4.51	9.04
	Root biomass	y = 0.212x^2^–8.186x + 97.39	0.979	7.09	16.53
	Shoot biomass	y = 0.277x^2^–10.38x + 94.46	0.944	4.93	9.67
Theaspirane	Germination	y = 0.007x^2^–0.754x + 97.93	0.660	o.r.	o.r.
	Root length	y = -0.123x^2^ + 3.640x + 102.4	0.668	*40*.*19*	*44*.*61*
	Shoot length	y = 0.123x^2^–5.415x + 97.92	0.977	12.27	o.r.
	Root biomass	y = -0.073x^2^ + 2.129x + 105.3	0.101	*45*.*73*	*51*.*75*
	Shoot biomass	y = 0.116x^2^–5.231x + 98.71	0.980	13.14	o.r.

r^2^_adj_ = adjusted coefficient of determination

IC_50_ = concentration that inhibits or reduces germination and growth at 50% of control

IC_80_ = concentration that inhibits or reduces germination and growth at 80% of control

o.r. = out of range

IC values calculated from equation overcoming the maximum assayed concentration are expressed in italics

In the case of *A*. *retroflexus* (Table 3), all the assayed compounds except theaspirane caused at least 50% inhibition of germination in the range of concentrations assayed, with verbenone achieving the lowest IC_50_ and IC_80_ values of 3.69 and 7.39 ppm, respectively. This compound, together with linalool and *α-*terpineol, also caused intense growth reductions below 25 ppm, the shoots being more sensitive than the roots.

The germination of *D*. *sanguinalis* was inhibited 50% by linalool, *α*-terpineol, verbenone, and eugenol (Table 4). The monoterpene *α-*terpineol was the most phytotoxic to *D*. *sanguinalis*, being able to inhibit germination 50% and 80% at 8.34 and 16.40 ppm in the Petri dish atmosphere, respectively. Eugenol, followed by linalool and verbenone were the most effective compounds in reducing seedling growth and biomass.

### Reversibility bioassays of the phytotoxic isolated VOCs

From the results obtained in the previous dose-response bioassays, linalool, terpinen-4-ol, *α*-terpineol, verbenone, and eugenol were assayed for reversibility at those concentrations that achieved a minimum of ten non-germinated seeds per replicate. The results of total germination of these pre-treated seeds incubated for 20 days in distilled water are represented in Table 5. Germination values are expressed as percentages relative to the control (non-pretreated seeds).

**Table 5:** Reversibility of the phytotoxic effects on the germination of two agricultural weed species pre-treated with different VOCs and then transferred to water. Data are expressed as percentages relative to the control ± SD.

Compound	Pre-treated concentration (ppm)	Germination (% ± S.D.)	
		*Amaranthus retroflexus*	*Digitaria sanguinalis*
Linalool	6.25	#	#
	12.5	#	#
	18.75	60.0 ± 14.1	2.6 ± 5.3
	25	67.5 ± 20.6	7.9 ± 10.1
Terpinen-4-ol	6.25	#	#
	12.5	#	#
	18.75	60.0 ± 8.2	5.3 ± 10.5
	25	45.0 ± 36.9	13.2 ± 13.2
*α-*Terpineol	6.25	#	26.3 ± 13.6
	12.5	60.0 ± 24.5	18.4 ± 17.9
	18.75	57.5 ± 20.6	7.9 ± 10.1
	25	62.5 ± 12.6	10.5 ± 14.9
Verbenone	6.25	52.0 ± 25.0	#
	12.5	42.5 ± 25.0	42.1 ± 14.9
	18.75	50.0 ± 16.3	13.2 ± 5.3
	25	40.0 ± 8.2	10.5 ± 8.6
Eugenol	6.25	#	#
	12.5	#	#
	18.75	78.9 ± 6.1	10.5 ± 8.6
	25	97.4 ± 10.1	5.3 ± 6.1

# Concentrations for which the minimum number of ten non-germinated seeds for the reversibility bioassay was not achieved

In the case of *A*. *retroflexus*, only the seeds pre-treated with verbenone or terpinen-4-ol at 25 ppm showed reversibility values below 50% after being transferred to distilled water, the effects of verbenone being the most persistent.

In contrast, the phytotoxic effects of the isolated VOCs were in general highly persistent on *D*. *sanguinalis* seeds. For all the VOCs, the germination percentages of *D*. *sanguinalis* seeds pre-treated with 18.75 and 25 ppm scored the lowest values of the bioassay, ranging between 2.6 and 13.2%.

For both target species and all compounds, the seeds that recovered the germination capacity produced seedlings with curved-yellowish roots, necrotic root tips, malformed calyptra, and/or prostrated shoots.

## Discussion

Following the methodology of Barney et al. [38], other authors have demonstrated the phytotoxic properties of different plant species (e.g., *Artemisia vulgaris*, *Calamintha nepeta*, *Acacia longifolia*) through the natural emission of volatile organic compounds in a closed atmosphere, e.g., [38, 48, 49]. However, the bioherbicide potential of the VOCs emitted by the aerial biomass of *U*. *europaeus and C*. *scoparius* is reported here for the first time. From our results, the flowering fresh plant material of both species can produce and emit volatile compounds able to inhibit at different extend the germination and/or early growth of two weed species. Both flowering branches and flowers of *U*. *europaeus* resulted phytotoxic to *A*. *retroflexus* and *D*. *sanguinalis* root growth and also reduced *A*. *retroflexus* shoot growth. Moreover, *C*. *scoparius* was able to inhibit significantly *D*. *sanguinalis* germination, and early growth of both target weeds, also exerting significant reduction of *D*. *sanguinalis* root biomass. The equivalent biomass quantity on a dry mass basis, composed of only flowers or flowering green branches, was able to produce similar phytotoxic effects, thus pointing out the potential of both species to produce and emit bioactive VOCs from the total aerial biomass. These results extend the interest of both shrub species as sources of natural products with bioherbicide potential, or to be used as bioherbicide green manures, as has been proposed for other allelopathic biomass-rich invasive species [36, 37].

The volatile profiles obtained by GC and GC/MS from both species and plant parts (flowering branches and flowers) gave us the qualitative and quantitative composition of VOCs that could be potentially released from them. Of course, the presence of a given compound in the volatile extract does not guarantee its natural release but reflects the potential for VOCs synthesis. In this sense, Cao et al. [21] and Boissard et al. [22] measured the emission fluxes of VOCs from *U*. *europaeus* as well as their seasonal variations. They found that branches from *U*. *europaeus* from England were large emitters of isoprene and the monoterpenes trans-ocimene and *α*-pinene, mainly over the hottest months from June to September, besides others such as camphene, sabinene, *β-*pinene, myrcene, limonene, and *γ*-terpinene to a lesser extent. In contrast, from our chemical profile of the volatile extract of *U*. *europaeus* from NW Spain gathered over spring blossom (April and May), just one monoterpene, the limonene derivative isomenthone, was detected. Otherwise, other not previously reported compounds were identified, i.e., two sesquiterpenes (*E*-nerolidol and 6, 10, 14-trimethylpentadecanone), one diterpene (phytol), two norisoprenoids (theaspirane A and B), and two benzenoids (eugenol and 4-vinyl-2-methoxyphenol). However, 89.91 and 61.12 percent of the volatile extract of flowering branches and flowers, respectively, was composed of aliphatic compounds. Of course, the environmental conditions such as different climate, geographical location, soil type, seasonality, phenological stage, and stress can underlie the different chemical compositions found in *U*. *europaeus*. It may also be possible that part of the VOCs from *U*. *europaeus* is largely emitted by the living plant, but otherwise completely or partially lost during harvesting, transportation, or processing. Moreover, they could be present but undetected, since 36% of the components were not identified in the flower volatile extract.

Because of their role in pollinator attraction, flowers are prone to be especially rich in allelochemicals. Cao et al. [21] found more than two-fold VOCs emission from flowering branches of *U*. *europaeus* than for branches without flowers, whereas López-Hortas et al. [16] found that the phenolic and volatile contents of flowers were higher than those in any other part of the plant. In our case, phytol, *E*-nerolidol, and 6, 10, 14-trimethylpentadecanone, where the main terpenoid constituents of the volatile extract of *U*. *europaeus* flowers. As this species cannot set seeds in the absence of active pollination [50], it is possible that some of those three terpenoids (i.e., *E*-nerolidol [51]) together with the other abundant aliphatic compounds found in the flowers extract, may play a role in pollinators attraction in *U*. *europaeus*, as has been discussed for the essence of flowers of other species [52] including Leguminous [53]. Nonetheless, we identified other VOCs in the extract of flowering branches of *U*. *europaeus* which were not present in the flower extract, i.e., isomenthone, theaspirane, eugenol, 4-vinyl-2-methoxyphenol, and certain aliphatic compounds. These compounds produced by vegetative parts (shoots, foliage or leaf spines) have been argued to play a bioactive role in plant defense [54, 55]. If naturally emitted, these VOCs should also be responsible for the phytotoxic effects on weeds observed in the volatile bioassays with flowering branches, since some of them have reputed bioherbicide effects (e.g., eugenol, [56, 57]) or have been isolated from phytotoxic essential oils [48].

The richness of active principles of *C*. *scoparius* and other species of the genus *Cytisus* is well known, and their wide traditional pharmacological uses ‘support the further application and exploitation for new drug development’ [12]. However, differently from other groups of natural compounds (i.e., alkaloids, phenolic acids, flavones, flavonols, and isoflavones), only one previous report dealt with the analysis of the volatile profile of *C*. *scoparius* flowers [58]. Kurihana and Kikuchi [58] detected a discrete number of VOCs by GC/MS in the essential oil of fresh flowers of *C*. *scoparius* from Japan. Some of them, as 1-octen-3-ol, palmitic, lauric and myristic acids, and linalool, also occurred in our chemical profile; others like isovaleranol, guaiacol, benzoic acid, cresols, and eugenol were not detected in our volatile extract. In our case, the flowering branches and flowers of *C*. *scoparius* from NW Spain yielded a high percentage of aliphatic compounds (>82%) and were rich in monoterpenes, oxygenated monoterpenes being quite abundant (>10%). Here, the monoterpene hydrocarbons *α*-pinene, *α*-terpinene, *γ*-terpinene, and the oxygenated monoterpenes terpinen-4-ol, verbenol, *α-*terpineol, verbenone, and *p*-menth-1,5-diene-8-ol were identified for the first time in *C*. *scoparius*.

Whereas *U*. *europaeus* presents some plasticity to regenerate vegetatively [6, 50], *C*. *scoparius* lacks the capacity for vegetative reproduction. So, since Scotch broom is an obligate outcrossing species [59], the investment of secondary metabolites to ensure pollination should be higher, even more diverse to attract a wider diversity of pollinators [55] over a single and shorter flowering period. In fact, some of the terpenoids identified in the floral extract have been reported to be involved in pollinators’ attraction ([51, 52], and references therein), including verbenone [60]. These compounds can be responsible for the significant to highly significant phytotoxic effects described for the volatile bioassays with *C*. *scoparius* flowering branches and flowers, as linalool, terpinen-4-ol or *α*-terpineol have well known phytotoxic activity, e.g., [34, 61]. Also, verbenone, a semiochemical with reputed pest deterrent properties because of its ecological role as anti-aggregation pheromone (see [62] as a review), has very recently been explored for its phytotoxicity [63]. Verbenone is synthetically obtained from the abundantly available pinenes in pine oleoresins, and then commercially used in tree protection [64]. This oxygenated monoterpene is also a particularly attractive starting material for the synthesis of the antitumoral diterpene Taxol (Bristol-Myers Squibb Company, Princeton, NJ). Verbenone is quite abundant in flavor complexes of edible aromatic species such as strawberry, raspberry, dill, rosmarinus, and spearmint ([64] and references therein). The natural occurrence of verbenone in the abundant biomass of *C*. *scoparius*, described here for the first time, extends the interest of this species for the exploitation of its natural active principles.

From the twelve compounds selected to be tested isolatedly for their phytotoxicity on the germination of the target agricultural weed species *A*. *retroflexus* and *D*. *sanguinalis*, the aliphatic compounds *n-*nonadecene, *n-*eicosane, *n-*heneicosane, *n-*docosane, *n-*tricosane and *n-*tetracosane were innocuous to *A*. *retroflexus* and *D*. *sanguinalis* germination and early growth at any concentration assayed. So, despite the high percentages of the aliphatic compounds in the volatile extracts of both shrub species, they seemed to have a limited contribution to the phytotoxicity observed in the volatile assays. The low phytotoxicity of the natural aliphatic compounds if compared with the aromatic ones is well known [65], as well as the stronger phytotoxic properties of terpenes compared with other VOC classes [66].

From the results obtained from the dose-response *in vitro* bioassays, the other six minority compounds (linalool, terpinen-4-ol, *α*-terpineol, verbenone, eugenol, and theaspirane) showed different degrees of phytotoxicity on the germination and early growth of both target species, even at very low concentrations.

Monoterpenes are argued to be involved in allelopathic processes as effective inhibitors of seed germination and seedling growth [67, 68], oxygenated monoterpenes being more bioactive than non-oxygenated [34, 69]. As a background of our work, the presence of high concentrations of linalool and *α*-terpineol in the Petri dish atmosphere was shown to prevent the germination and growth of both *A*. *retroflexus* and *D*. *sanguinalis* [70]. Kordali et al. [69] reported that linalool, *α*-terpineol, and terpinen-4-ol at 100 ppm completely inhibited seed germination of *A*. *retroflexus*. Also, eugenol sprayed at 1.5% (v/v) on *A*. *retroflexus* seedling caused loss of membrane integrity and inhibited growth severely [71]. Lower concentrations of linalool, terpinen-4-ol, and eugenol were shown to inhibit the germination of the model species *Lactuca sativa* [34] or the early growth of *Cassia occidentalis* and *Bidens pilosa* [57]. From the results obtained from our dose-response bioassays, verbenone, linalool, and *α*-terpineol seemed to be responsible for the phytotoxicity observed in *C*. *scoparius*, terpinen-4-ol showing more moderate phytotoxic effects on both *A*. *retroflexus* and *D*. *sanguinalis*, with higher IC values. Following the trend observed in the volatile bioassay with plant material, shoot growth was generally more sensitive than root elongation to the effects of the isolated VOCs, with lower IC_50_ and IC_80_ values for shoot length and biomass.

Noteworthy, verbenone was able to inhibit 80% germination of *A*. *retroflexus* and 50% root and shoot length of *D*. *sanguinalis* at less than 9 ppm in the Petri dish atmosphere. As far as we know, only two recent studies dealt with the phytotoxicity of verbenone added to the culture solution [63, 72]. These studies showed that verbenone applied at 100 to 150 μg mL^-1^ exerted extremely weak inhibition of the seedling growth of *Echinochloa crus-galli* and *Brassica campestris* [63], or just a delay on *L*. *sativa* germination [72]. We explain such discrepancy of results by the relative insolubility of VOCs in water [73], which could have prevented verbenone to reaching the target seeds when added to an aqueous culture solution, whereas being highly phytotoxic when volatilized in the Petri dish atmosphere.

The richness and relative abundance of oxygenated monoterpenes in the volatile profile of *C*. *scoparius* may underlie the higher phytotoxic effects observed of its flowering biomass if compared with *U*. *europaeus*, being able to inhibit the root and shoot growth of both weed species. Note that linalool, which could control by 50% the germination and early growth of both dicotyledon and monocotyledon agricultural weed species at very few ppm, was detected only in the volatile extract of the flowering branches, but not in the flowers alone, as was the case for *α*-terpineol. In the same direction, the benzenoid eugenol, which was extremely effective in controlling *A*. *retroflexus* and *D*. *sanguinalis* shoot and root growth- with the lowest IC_80_ values-, was only found in the flowering branches of *U*. *europaeus*, but not in the flowers extract, just like the oxygenated norisoprenoid theaspirane, which was moderately phytotoxic to *D*. *sanguinalis* shoot growth. These findings extend the interest of Scotch broom and gorse as natural sources of phytotoxins also outside their flowering periods, and also to be used both together as bioherbicide biomass under a practical point of view.

The study of the reversibility of the phytotoxic effects of linalool, terpinen-4-ol, *α*-terpineol, verbenone, and eugenol also threw quite exciting results. The toxic effects were highly persistent on *D*. *sanguinalis*, since the seeds that had been exposed to 18.75 to 25 ppm of any of the five bioassayed VOCs were not able to recover more than 13.5% mean germination. Noteworthy, those seeds that reversed the germination inhibition produced damaged seedlings with similar symptoms than those exposed to the phytotoxic VOCs during germination and early growth but additionally showed prostrated shoots applied to the wet filter paper. So that, even when the weed seeds subjected to inhibitory concentrations could recover germination capacity in different degree, we can consider the effects on the embryo as permanent or at least persistent [74, 75], because of producing unviable seedlings even after removing the phytotoxin.

Finally, reconsidering the naturally emitted VOCs bioassays, we must bear in mind that the volatile extracts of the shrub species rendered a mean yield of 0.06% (w/w, on a fresh mass basis) with a mean density of 0.85 g mL^-1^. That little yield, if related to the fresh flowering biomass used in the volatile assay, corresponds to ca. 4.2 to ca. 4.5 μL of volatile extract in each of the 1 L chambers for *U*. *europaeus* and *C*. *scoparius*, respectively. So, as an example, the conspicuous phytotoxic effects on the weeds germination and seedling growth produced by the flowering branches of *C*. *scoparius* in the volatile assay were caused by, at most, the joint action of only ca. 0.14, 0.12, 0.06, and 0.09 ppm of linalool, terpinen-4-ol, *α*-terpineol, and verbenone, respectively, among other VOCs. Obviously, the real concentration of VOCs emitted by the plant material into the test chamber must be even lower. Considering the dose-response curves of each bioassayed VOC and the obtained IC_50_ values, the phytotoxicity observed in the volatile bioassays had to be due not to one VOC in particular, but to the combined action of minority components [38, 76], or to the interactions among some of them and other non-studied compounds present in the volatile extract. In fact, the synergistic phytotoxic action of the open-chain alcohol linalool and the monocyclic one terpinen-4-ol have been described in the literature [34, 77]. Also, we do not discard possible synergies or antagonisms with the abundant aliphatic compounds, if emitted by the plant material, and other phytotoxic VOCs taking part in the profile, interactions which are worth studying. Approaches that use headspace trapping techniques to collect and concentrate the volatiles released into the airspace, combined with GC/MS, would through light on which VOCs are responsible for the phytotoxicity observed in these naturally emitted volatile bioassays, as well as their real concentrations in the controlled atmosphere.

## Conclusions

The bioherbicide potential of the legume shrub species *Ulex europaeus* and *Cytisus scoparius* is reported in this work for the first time. The flowering fresh plant material of both species can produce and emit volatile compounds able to inhibit at different extend the germination and/or early growth of two agricultural weeds: *Amaranthus retroflexus* and *Digitaria sanguinalis*. Complete VOCs profiles from volatile extracts of *U*. *europaeus* and *C*. *scoparius* were obtained by GC and GC/MS. From these novel profiles, both species receive re-energized attention as sources of bioactive compounds. Particularly, *C*. *scoparius* revealed as rich in oxygenated monoterpenes as terpinen-4-ol, *α*-terpineol, and verbenone, as well as the previously reported linalool. Theaspirane and eugenol, among others, are also described for the first instance in *U*. *europaeus*.

Using dose-response bioassays with pure compounds, these VOCs were argued to be involved in the phytotoxicity observed for the plant materials, even at very low concentrations. The seeds of the agricultural weeds exposed to these VOCs produced damaged seedlings, were unable to recover germination capacity after removing the phytotoxin or, when recovered, produced unviable seedlings.

Our results extend the interest of the abundant *U*. *europaeus* and *C*. *scoparius* for the obtention of natural products with bioherbicide potential, or to be used as allelopathic biomass in the development of new sustainable agricultural practices. Further studies are required to evaluate the herbicide effectiveness under realistic field approaches, in which soil could compromise the persistence of phytotoxicity, as well as the putative side-effects on crops.

## Supporting information

S1 Fig Scheme of the *in vitro* volatile bioassay. Schematic representation of the experiments carried on seeds and seedlings of the weed species *Amaranthus retroflexus* and *Digitaria sanguinalis*.
(TIF)

S2 Fig Dose-response curves of six VOCs on the germination and early growth of *Amaranthus retroflexus*. Mean values are represented as percentages relative to the control. Error bars represent standard deviation (SD). Mean values labelled with distinct letters are significant different at *P* ≤ 0.05 (ANOVA or Kruskal-Wallis H test).
(TIF)

S3 Fig Dose-response curves of six VOCs on the germination and early growth of *Digitaria sanguinalis*. Mean values are represented as percentages relative to the control. Error bars represent standard deviation (SD). Mean values labelled with distinct letters are significant different at *P* ≤ 0.05 (ANOVA or Kruskal-Wallis H test).
(TIF)

S1 Table *P*-values obtained for the two-way ANOVA of the effects of the aliphatic VOCs (*n*-nonadecane, *n*-eicosane, *n*-heneicosane, *n*-docosane, *n*-tricosane and *n*-tetracosane), the concentration assayed, and their interactions, on the germination of the weed species *Amaranthus retroflexus* and *Digitaria sanguinalis*. (DOCX)

S2 Table Effects of volatile aliphatic compounds at different concentrations on the germination of the weed species *Amaranthus retroflexus* and *Digitaria sanguinalis*. (DOCX)
