# Supplementary figures and images for: On the bioherbicide potential of Ulex europaeus and Cytisus scoparius: Profiles of volatile organic compounds and their phytotoxic effects
Source: PLoS One. 2018 Oct 29;13(10):e0205997. doi: 10.1371/journal.pone.0205997 (PMC6205617; doi:10.1371/journal.pone.0205997)

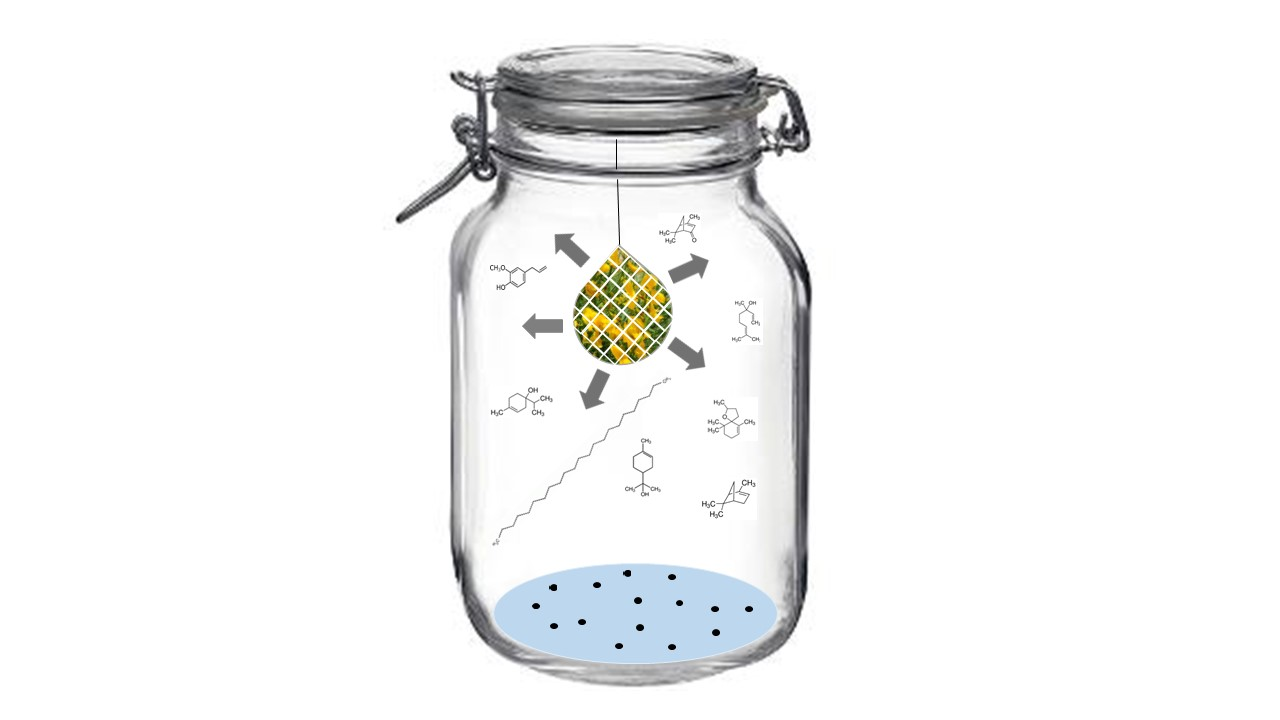

Supplement: S1 Fig — Schematic representation of the experiments carried on seeds and seedlings of the weed species Amaranthus retroflexus and Digitaria sanguinalis. (TIF) [file pone.0205997.s001.tif]

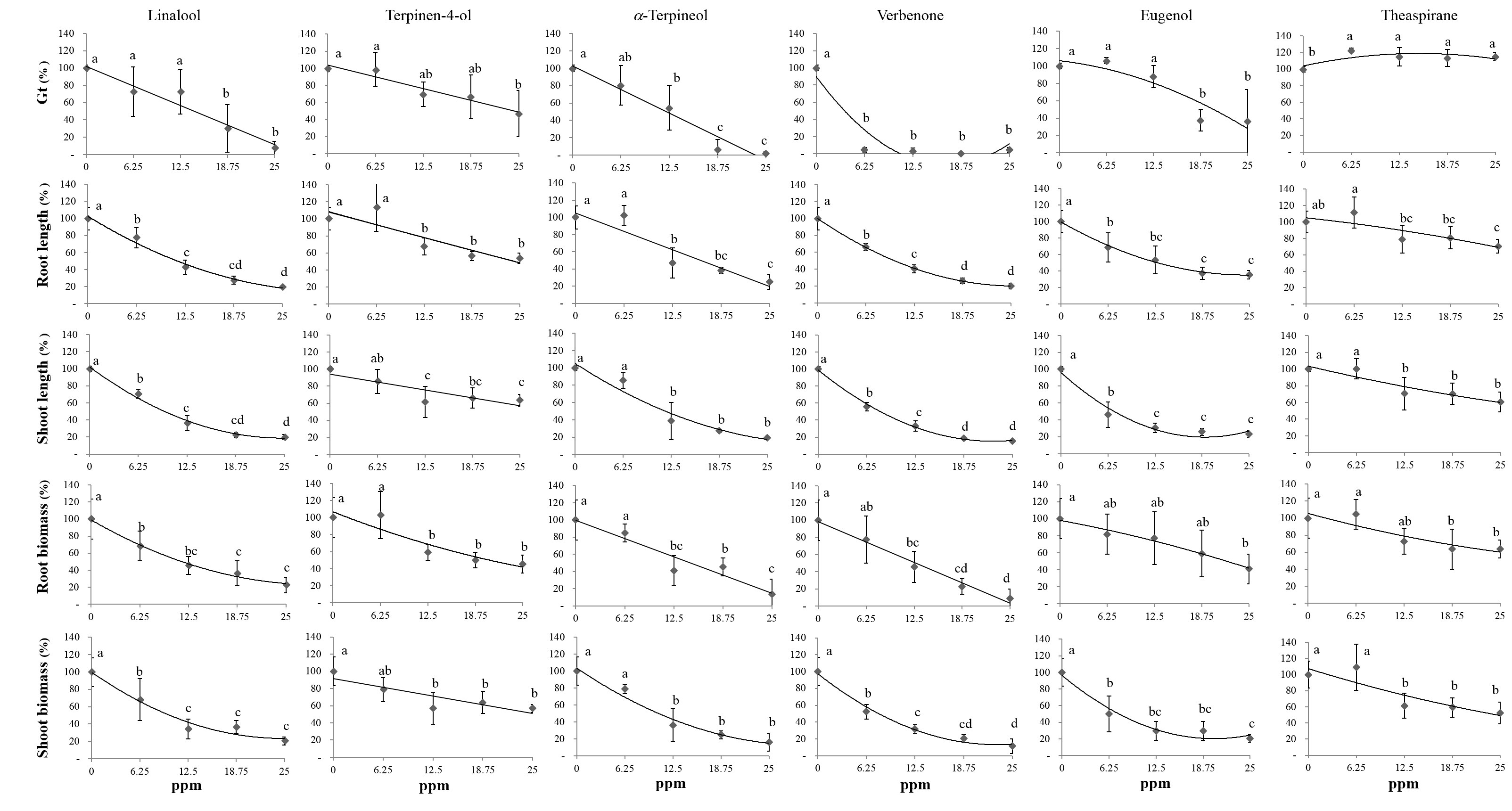

Supplement: S2 Fig — Mean values are represented as percentages relative to the control. Error bars represent standard deviation (SD). Mean values labelled with distinct letters are significant different at P ≤ 0.05 (ANOVA or Kruskal-Wallis H test). (TIF) [file pone.0205997.s002.tif]

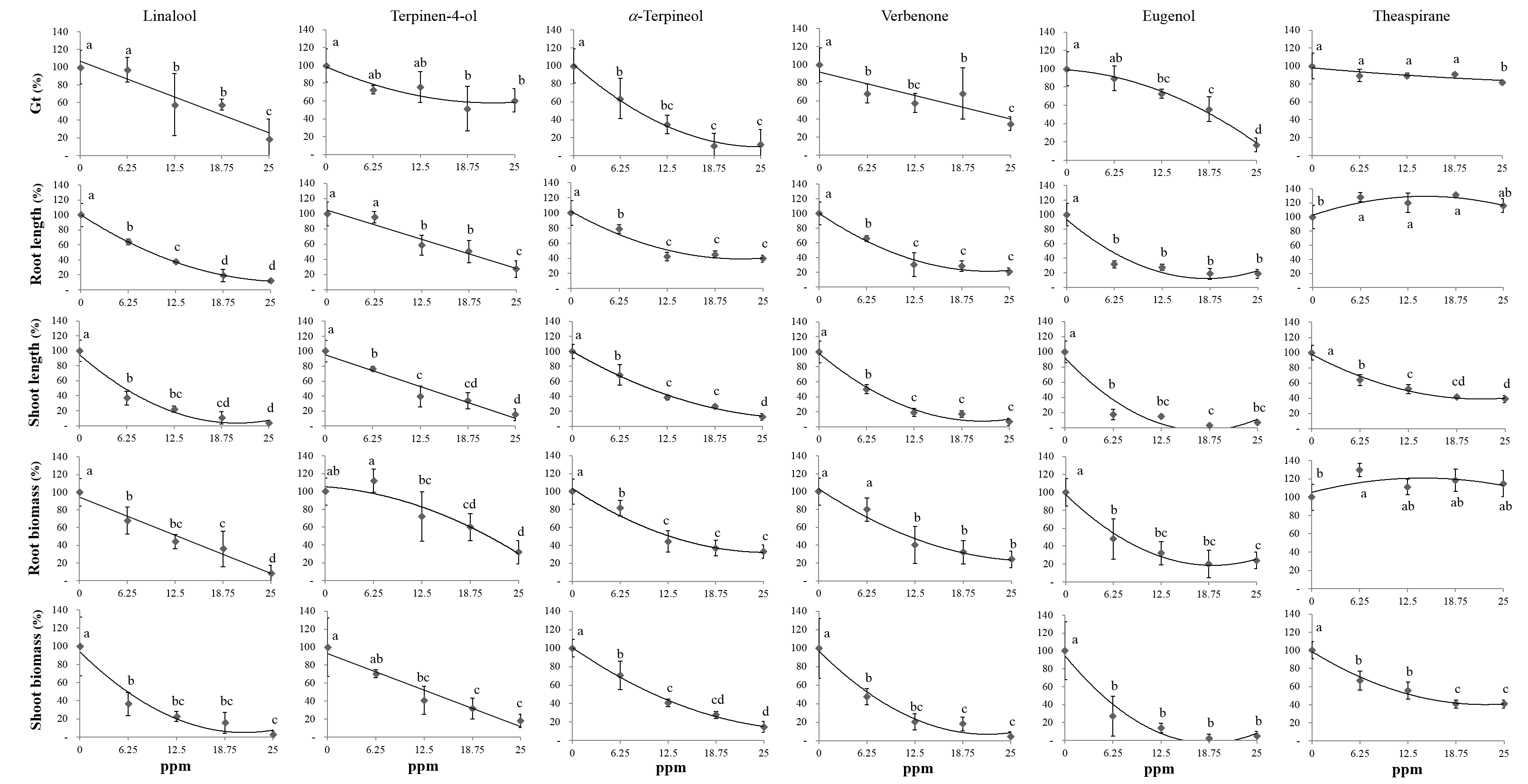

Supplement: S3 Fig — Mean values are represented as percentages relative to the control. Error bars represent standard deviation (SD). Mean values labelled with distinct letters are significant different at P ≤ 0.05 (ANOVA or Kruskal-Wallis H test). (TIF) [file pone.0205997.s003.tif]
