# Supplementary material for: On the bioherbicide potential of Ulex europaeus and Cytisus scoparius: Profiles of volatile organic compounds and their phytotoxic effects
Source: PLoS One. 2018 Oct 29;13(10):e0205997. doi: 10.1371/journal.pone.0205997 (PMC6205617; doi:10.1371/journal.pone.0205997)
Supplement: S1 Table — (DOCX) [file pone.0205997.s004.docx]

**S1 Table. *P*-values obtained for the two-way ANOVA of the effects of the aliphatic VOCs (*n-*nonadecane, *n-*eicosane, *n-*heneicosane, *n-*docosane, *n-*tricosane and *n-*tetracosane), the concentration assayed, and their interactions, on the germination of the weed species *Amaranthus retroflexus* and *Digitaria sanguinalis*.**

|  | | Compound | Concentration | Compound × Concentration |
| --- | --- | --- | --- | --- |
| *Amaranthus retroflexus* | Germination | 0.819 | 0.744 | 0.858 |
| *Digitaria sanguinalis* | Germination | 0.552 | 0.306 | 0.409 |

Effects of treatments significant at *P* ≤ 0.05, very significant at *P* ≤ 0.01, highly significant at *P* ≤ 0.001, and not significant at *P* > 0.05
