# Supplementary material for: On the bioherbicide potential of Ulex europaeus and Cytisus scoparius: Profiles of volatile organic compounds and their phytotoxic effects
Source: PLoS One. 2018 Oct 29;13(10):e0205997. doi: 10.1371/journal.pone.0205997 (PMC6205617; doi:10.1371/journal.pone.0205997)
Supplement: S2 Table — (DOCX) [file pone.0205997.s005.docx]

**S2 Table. Effects of volatile aliphatic compounds at different concentrations on the germination of the weed species *Amaranthus retroflexus* and *Digitaria sanguinalis*.**

| Compound | Pre-treated  concentration  (ppm) | Germination (% ± S.D.) | | | |
| --- | --- | --- | --- | --- | --- |
|  |  | *Amaranthus retroflexus* | | *Digitaria sanguinalis* | |
|  |  |  | *P*-value |  | *P*-value |
| *n-*Nonadecane | 0 | 100.0 ± 7.4 | 0.330 | 100.0 ± 12.2 | 0.115 |
|  | 6.25 | 93.4 ± 14.5 |  | 106.1 ± 20.0 |  |
|  | 12.5 | 100.0 ± 15.5 |  | 91.84 ± 13.9 |  |
|  | 18.75 | 100.0 ± 15.5 |  | 124.5 ± 13.9 |  |
|  | 25 | 101.3 ± 7.9 |  | 102.0 ± 20.5 |  |
| *n-*Eicosane | 0 | 100.0 ± 7.4 | 0.463 | 100.0 ± 12.2 | 0.672 |
|  | 6.25 | 96.1 ± 6.6 |  | 114.3 ± 33.3 |  |
|  | 12.5 | 96.1 ± 12.4 |  | 110.0 ± 10.5 |  |
|  | 18.75 | 100.0 ± 8.6 |  | 102.0 ± 14.1 |  |
|  | 25 | 102,6 ± 5.3 |  | 108.2 ± 24.4 |  |
| *n-*Heneicosane | 0 | 100.0 ± 7.4 | 0.470 | 100.0 ± 12.2 | 0.693 |
|  | 6.25 | 96.0 ± 10.8 |  | 112.2 ± 25.3 |  |
|  | 12.5 | 100.0 ± 14.3 |  | 112.2 ± 13.9 |  |
|  | 18.75 | 84.2 ±18.2 |  | 112.2 ± 7.8 |  |
|  | 25 | 101.3 ± 18.4 |  | 102.0 ± 15.6 |  |
| *n-*Docosane | 0 | 100.0 ± 7.4 | 0.946 | 100.0 ± 12.2 | 0.272 |
|  | 6.25 | 109.2 ± 13.2 |  | 87.8 ± 19.3 |  |
|  | 12.5 | 105.3 ± 6.1 |  | 100.0 ± 12.2 |  |
|  | 18.75 | 93.4 ± 18.9 |  | 97.9 ± 20.0 |  |
|  | 25 | 94.7 ± 18.7 |  | 102.0 ± 8.2 |  |
| *n-*Tricosane | 0 | 100.0 ± 7.4 | 0.909 | 100.0 ± 12.2 | 0.199 |
|  | 6.25 | 98.7 ± 9.9 |  | 112.2 ± 10.3 |  |
|  | 12.5 | 92.1 ± 24.9 |  | 124.5 ± 23.5 |  |
|  | 18.75 | 96.5 ± 13.3 |  | 93.9 ± 31.6 |  |
|  | 25 | 94.7 ± 12.2 |  | 89.8 ± 31.9 |  |
| *n-*Tetracosane | 0 | 100.0 ± 7.4 | 0.765 | 100.0 ± 12.2 | 0.861 |
|  | 6.25 | 100.0 ± 8.6 |  | 118.4 ± 4.7 |  |
|  | 12.5 | 94.7 ± 14.9 |  | 102.0 ± 14.1 |  |
|  | 18.75 | 97.4 ± 3.0 |  | 110.2 ± 14.1 |  |
|  | 25 | 88.2 ± 5.0 |  | 95.9 ± 12.2 |  |

Values denote mean ± S.D. Data given as percentage with respect to their controls. For each species, *P*-values of the effects of treatments are significant at *P* ≤ 0.05, very significant at *P* ≤ 0.01, highly significant at *P* ≤ 0.001, and not significant at *P* > 0.05 (ANOVA or Kruskal-Wallis *H* test).
